# Most suitable plant communities for the slope reclamation of the Zhengzhou-Xinxiang section of the Beijing-Hong Kong-Macao expressway

**DOI:** 10.1371/journal.pone.0297004

**Published:** 2024-02-14

**Authors:** Wei Cao, Xiaoqi Wu, Niuniu Zhu, Zhenyu Meng, Chenxi Lv, Xi Li, Guojie Wang

**Affiliations:** 1 Henan Institute of Science and Technology, Xinxiang, Henan, China; 2 Henan Province Engineering Research Center of Horticultural Plant Resource Utilization and Germplasm Enhancement, Xinxiang, Henan, China; 3 Department of Plant Science, The Pennsylvania State University, PA, United States of America; Central University of Haryana School of Life Sciences, INDIA

## Abstract

The construction of expressways in China has produced diverse habitats along slopes characterized by steep gradients, uneven water distribution, poor soil conditions, and no routine maintenance. Manually planting beneficial species is an essential method of effectively improving slope soils to prevent soil erosion. However, few studies have evaluated the reclamation effects and plant community composition and structure used to restore slopes along expressways. This study focused on the Zhengzhou-Xinxiang section of the Beijing-Hong Kong-Macao Expressway. A total of 10 representative plant communities were evaluated using the analytic hierarchy process (AHP)–fuzzy integrated evaluation method. The sites were divided into four layers, namely, plant communities, soil nutrients, soil physical properties, and other ecological factors, and 14 indicators were assessed. The evaluation results showed that four of these plant communities (PCs) were excellent, three PCs were good, one PC was normal, two PCs were poor. The four excellent PCs had high Shannon-Wiener index, pielou index, richness index or community productivity. It is worth noting that most excellent plant community structures were tree + shrub + herb. Based on these results, we recommend that fill slopes should be restored using a combination of trees, herbs, and shrubs; also, the vegetation should include native plants, such as *B*. *papyrifera*, *U*. *pumila*, *A*. *fruticosa*, and *Cynodon dactylon* (L.). This study could provide ideas for plant community composition and structure of new highway slopes in similar climate environment, and provide theoretical support for plant community composition and structure and soil improvement for the existing slope.

## Introduction

China has experienced unprecedented economic development in recent decades, which has led to the rapid construction of expressways and the formation of dense highway networks. The construction of expressways has provided people with many conveniences; it also promoted economic and cultural exchange and development among the various regions of China.

Slopes along roadsides present varying gradients, which has led to the formation of unique habitats along wide highway roadbeds, including extensive excavation and fill sites [1]. For every 1 km of expressway constructed in China, approximately 50 to 70,000 m^2^ of bare slope area is formed, with an annual value of about 200 to 300 million m^2^. The ecological issues associated with barren slopes (such as severe landscape fragmentation, habitat destruction, and soil erosion) can develop into serious road safety problems [2, 3]. Once an expressway slope is damaged, the ecological recovery rate is prolonged due to the poor slope soil, fragmented geographical location and climatic constraints.

Research on roadside slopes in China is relatively recent, and most of the earlier studies focused on improving the safety of slope protection structures, such as increasing slope stability through slope protection engineering [4–6] or performing slope ecological restoration using vegetated concrete [7]. Increasing awareness of environmental protection has increased research on improving the reclamation of roadside slope plant communities; moreover, the potential of roadside slope revegetation design has shifted from simple regreening to sustainable slope reclamation [8]. For example, Wang et al. [9] examined the significant variations in soil respiration and soil hardness among plant community combinations to determine the best plant community combination for the ecological reclamation of slopes. Cao et al. [10] designed a runoff field on a highway slope with the optimum plant community combination identified through experimental data. Scholars have also suggested that plant communities should be configured to include trees, shrubs, and grasses that are drought- and cold-resistant and thus show high environmental adaptability [11]. Research has also revealed that combining legumes with perennial herbs benefits slope reclamation [12].

These recent theories have greatly improved the ecological reclamation of highway slopes in China by enhancing soil and water conservation, especially in the early stages of slope reclamation, when plants initially grow vigorously [13]. However, as the slope community is established over time, some initially planted target species are successively lost, which greatly alters the plant community structure. Vegetation reclamation can easily become homogeneous, thus hindering the formation of a stable plant community [14]. Relevant research generally focuses on the early reclamation stage. However, most roads in China were constructed over 20 years ago, which means that slope reclamation effectiveness, plant community composition and diversity, and soil characteristics have not been investigated since the initial reclamation effort. Therefore, evaluating the effect of ecological reclamation on slopes after the initial reclamation period is very important [15]. Research can guide improvements in existing slope landscapes and provide a theoretical basis for plant configurations on new slopes. Henan Province is a major highway development province in China and will have over 10,000 km of highways based on the 14th Five-Year Plan. This plan calls for over 3,000 km of new highways to realize the "city-city ring road" and "county-county double highway" initiatives. Therefore, the characteristics of slope plant communities that were restored over 20 years ago must be investigated, and the reclamation effects of different plant communities must be determined to guide successful plant configurations on new slopes. The Zhengzhou-Xinxiang (Zheng-Xin) section of the Beijing-Hong Kong-Macao Expressway represents one of the earlier highways in Henan Province, and it was restored over 23 years ago. Since then, the plant community species composition and community structures have changed significantly from a single shrub community of *Amorpha fruticosa* (2 to 4 shrubs/m^2^) in the early years to a diverse array of community structures, including tree + shrub + grass, tree + grass, and shrub + herb.

We combined fuzzy hierarchical analysis with fuzzy comprehensive to evaluate the ecological reclamation of slopes [16]. Representative plant communities along the slopes of the Zheng-Xin section of the Beijing-Hong Kong-Macao Expressway were used to evaluate the effect of plant reclamation along slopes. The results will provide guidance for slope landscape improvements and theoretical support for the configuration of new slope plant communities in similar environments to promote successful slope ecological reclamation. We effectively supplement the theories of slope plant communities and soil characteristics in older reclamation areas (over 20 years old).

## Materials and methods

### Study area

**Fig 1:**
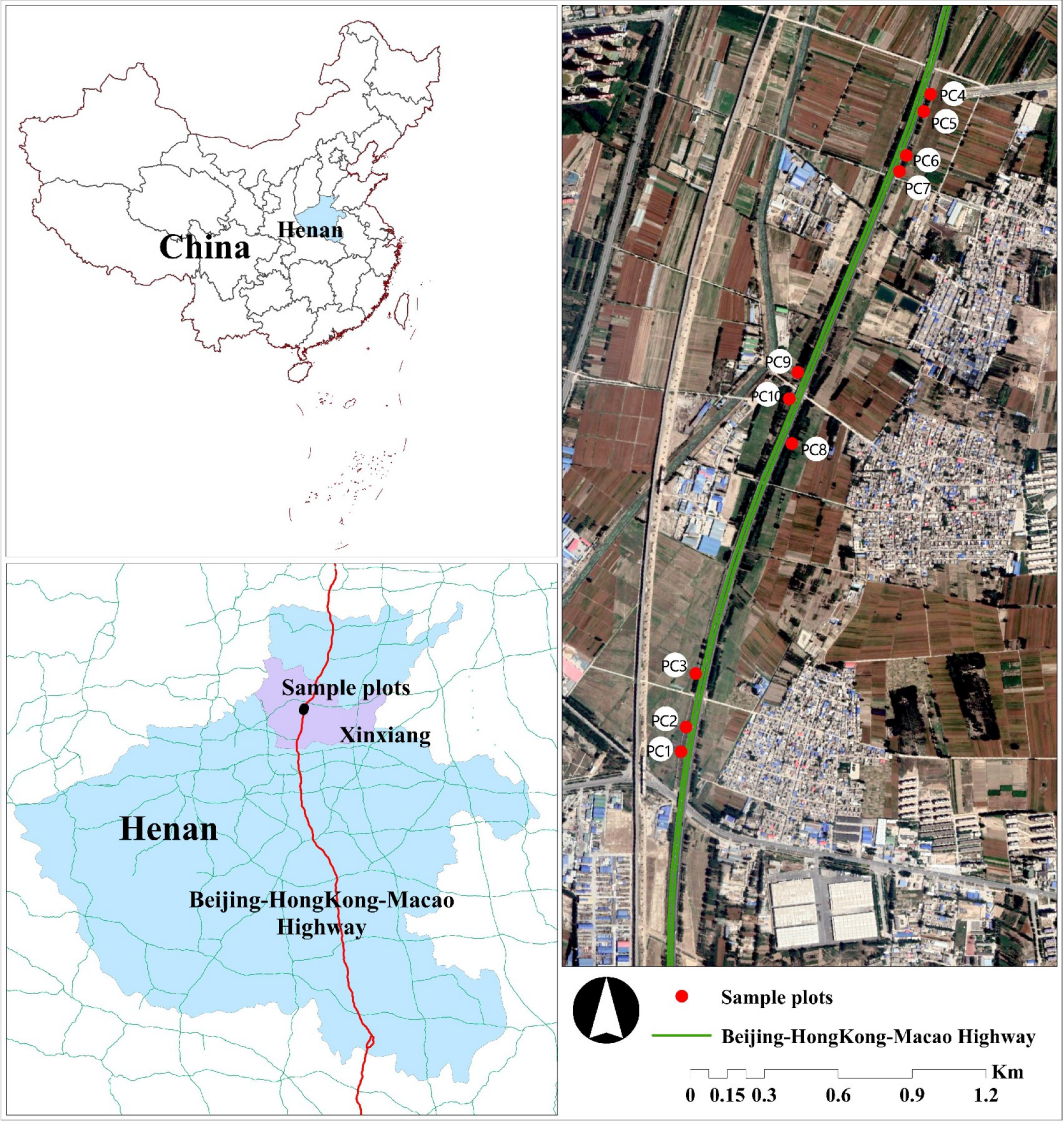
Location map.

The research location was within the Henan section of the Beijing-Hong Kong-Macao Expressway—which opened to traffic in 1997—in the southeastern section of Xinxiang city. The slope protection infrastructure uses an arch-shaped frame [17]. The area has a north temperate continental climate with cold winters and hot summers, four distinct seasons, an average temperature of 14°C, annual precipitation of 573.4 mm, and a wind pattern dominated by southeast winds [18]. The survey section is about 33 km, the common vegetation communities on the slope are *A*.*fruticosa*, *B*.*papyrifera* and *U*.*pumila*. Most of the *B*.*papyrifera* and *U*.*pumila* vegetation communities are concentrated in Xinxiang city. However, the community structure of these three common plant communities is different. Therefore, ten representative plant communities are selected as the research subjects (Fig 1).

### Plant sampling and species diversity assessment

#### Plant community survey

**Fig 2:**
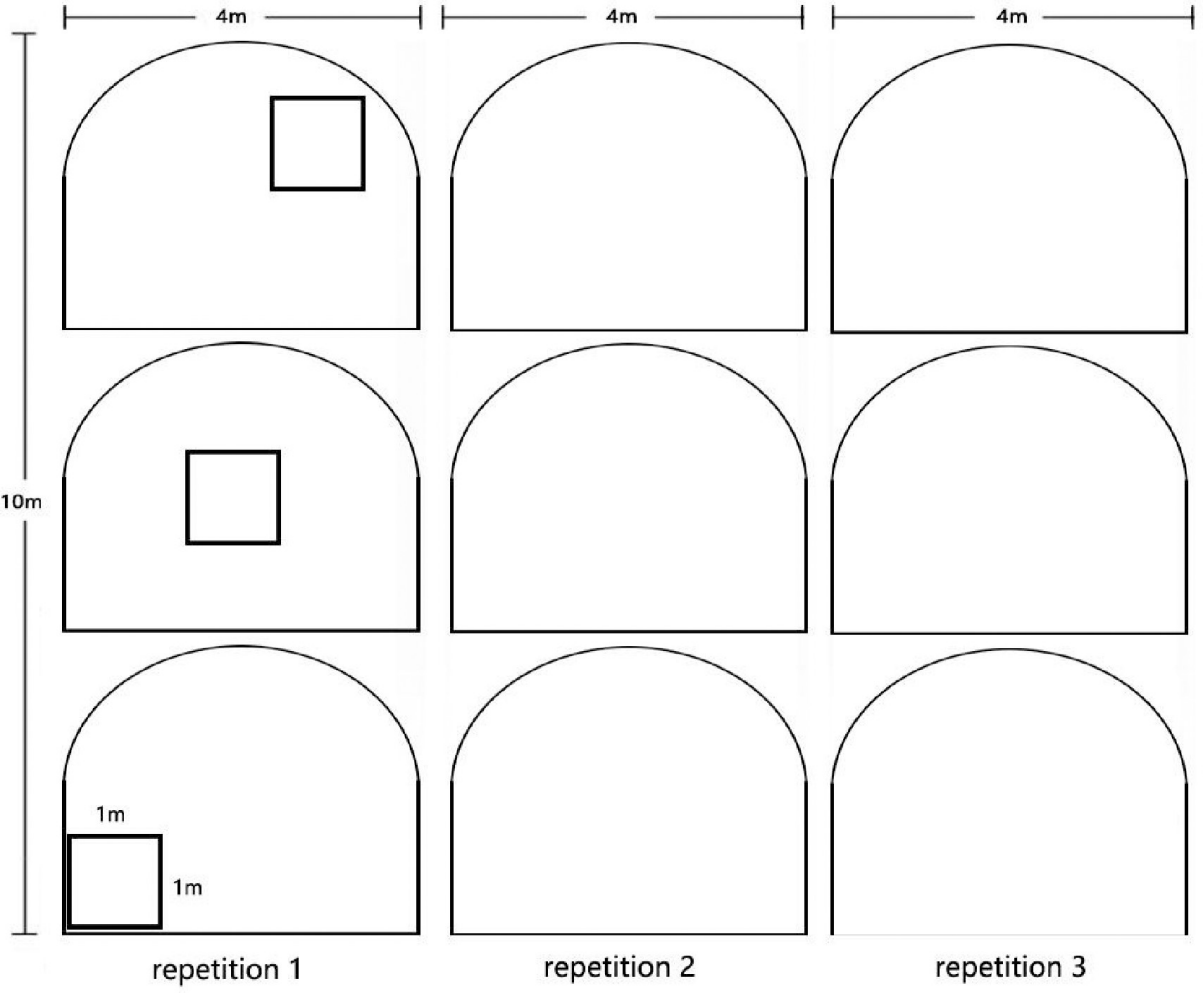
Sample plots setting.

In October 2020, the slope plant communities of the Zheng-Xin section of the Beijing-Hong Kong-Macao Expressway were investigated and sampled. Ten habitats were selected for a detailed investigation based on the results of the general survey. In each habitat (10) 3 plots (replicates) were surveyed (10×3) for trees, the same for herbs [19] (Fig 2). The plant communities were named according to their geographical location and dominant species (Table 1).

**Table 1:** Names of the plant communities and sample sites of the study section.

Number of PC^a^	Name of plant community	Topographic aspect	Slope gradient
**PC 1**	Guandi^b^ *Ulmus pumila + Amorpha fruticosa*	Western	46°
**PC 2**	Guandi A. *fruticosa + Artemisia annua*	Western	46°
**PC 3**	Guandi *Broussonetia papyrifera*	Western	32°
**PC 4**	Zhangdi^b^ *A*. *fruticosa*	Eastern	35°
**PC 5**	Zhangdi *A*. *fruticosa + Periploca sepium*	Eastern	35°
**PC 6**	Zhangdi *B*. *papyrifera + A*. *fruticosa*	Eastern	31°
**PC 7**	Zhangdi *U*. *pumila + A*. *fruticosa*	Eastern	31°
**PC 8**	Yuandi^b^ *B*. *papyrifera*	Eastern	30°
**PC 9**	Yuandi *B*. *papyrifera + Humulus scandens*	Western	31°
**PC 10**	Yuandi *A*. *fruticosa + Setaria viridis (L*.*) Beauv*	Western	31°

^a^PC is short for plant community.

^b^Guandi, Zhangdi and Yuandi are place names.

#### Species diversity calculation

The plant community compositon and structure was recored [19], and α-diversity were calculated. Indicators of community species diversity included the richness index (R), Shannon–Wiener index (H), and Pielou evenness index (E), which were calculated using the following equations [20, 21]:

Species richness: R = number of species in the sample plot

Shannon–Wiener index:

$$H=-\sum_{i=1}^{s}\;P_{i}\;\ln\;P_{i}$$ (1)

Pielou index:

E=H/lnS (2)

where S is the number of species in the sample; Pi is the proportional abundance of species; i is the base of the logarithm,

### Biomass determination

To determine plant productivity (i.e., biomass), tree biomass (W) was calculated by consulting biomass models and parameter tables based on the measured diameter at breast height and height for different tree species [22, 23]. To determine the plant productivity of shrubs and herbs, plants were harvested and placed in labeled experimental bags, dried in an oven at 80°C for 24 h, and then weighed [24]. The simulated equations for tree biomass are as follows:

$$W=a(D^{2}\;H)b$$ (3)

where W denotes biomass, H denotes tree height, and a and b are coefficients with values of 0.0315 and 0.9855, respectively. Both a and b are simulated equation coefficient values, not absolute values.

### Soil sampling and analysis

Each community sample plot was collected following an S-shape, and the soil was collected at a depth of 0–20 cm with three replicates. The soil samples were thoroughly mixed before determining the soil chemical properties. The investigated soil chemical properties included total nitrogen (TN), total phosphorus (TP), total potassium (TK), soil organic matter (SOM), and pH (pH). The physical properties of the soil were determined using the ring knife method with three replicates. The physical indexes included the soil saturation water holding capacity, soil moisture content, and soil bulk density [25]. TN was determined by the semi-micro Kjeldahl method. TP was determined by the hydrofluoric acid-molybdenum antimony anti-colorimetric method. TK was determined by the flame photometer method. SOM was determined by the potassium dichromate external heating method. Soil pH was determined by the potentiometric method [5, 26].

### Evaluation index system establishment

The effectiveness of the slope reclamation was evaluated using the hierarchical analysis–fuzzy integrated evaluation method. The hierarchical analysis method was proposed in the 1970s by A.L. Saaty, an American operations researcher [27]. In order to comprehensively evaluate the slope ecological reclamation effect, we not only considered the quantitative indicators of slope plant community and soil characteristics, but also added the qualitative indicators of ecological sustainability and ecological protection function for plant community. This method quantifies qualitative factors and calculates the hierarchal order using an analytic hierarchy process (AHP) evaluation method to derive weighted vectors of relevant factors; it is then combined with the fuzzy comprehensive evaluation method to establish an affiliation matrix. The affiliation degree and weight are then synthesized to scientifically evaluate the results [28–30].

The soil–plant complex of the slope was constructed to reflect the current status of the community (Table 2), and it involved four layers of criteria: plant community, soil nutrients, soil physical properties, and ecological factors. The plant community layer was evaluated based on the Shannon-Winener index, productivity, Pilou index, and Richness index. The soil nutrient layer was evaluated based on the SOM, TN, TP, TK, and pH. The soil physical property layer was evaluated based on the soil saturation water holding capacity, soil water content, and soil bulk density. The ecological factor layer was evaluated based on the community’s ecological functions and sustainability. There are eight index factors in soil nutrients and soil physical properties that are used to determine the performance of various soil functions. Organic matter in the soil determines the supply capacity for soil nutrients; TN, TP, and total potassium are important nutrients that affect plant growth and functions [31]; pH affects the availability of most soil nutrients, with a near-neutral pH generally improving soil nutrient uptake [32]; plant community sustainability and slope ecological protection functions contribute to our overall understanding of slope quality [33].

**Table 2:** AHP comprehensive evaluation index system.

Target layer	Guideline layer		Indicator layer	
**Most suitable plant communities for the slope reclamation of the Zhengzhou-Xinxiang section of the Beijing-Hong Kong-Macao Expressway** **A**	B1	plant community	C1	Shannon-Winener index of PC
			C2	PC productivity
			C3	Pielou index of PC
			C4	Richness of PC
	B2	Soil Nutrients	C5	SOM
			C6	TN
			C7	TP
			C8	TK
			C9	pH value
	B3	Soil physical properties	C10	Soil saturation water holding capacity
			C11	Soil moisture content
			C12	Soil bulk density
	B4	Ecological factors	C13	Sustainability of PC
			C14	Ecological protection function of PC

### Comprehensive evaluation model

#### Qualitative index evaluation criteria

Our evaluation was based on a review of relevant literature on the Delphi evaluation method and combined with feedback from 20 experts. The percentage statistics method added the evaluation results. A set of rubrics was established for the two qualitative indicators, plant community sustainability and ecological protection functions of plant community, and the evaluation criteria for the corresponding classification levels were clarified.

#### Quantitative index evaluation criteria

We collated all relevant literature and expert opinions. The field research data were used to identify the optimum quantitative indexes for evaluating the grading criteria when the index characteristics were combined. The soil evaluation grading involved analyzing the 2nd soil nutrient grading in China [34, 35]. Plant community productivity was calculated after an extensive literature review of productivity models with related tree species (Table 3).

**Table 3:** Comprehensive evaluation model for highway slope reclamation.

Guideline layer		Meaning of each grade		Excellent	Good	Normal	Poor
**B1**	**PC**	C1	Shannon-Winener index of PC	2–1.8	1.8–1.5	1.5–1	1–0.5
		C2	PC productivity	>80000	60,000–75,000	40,000–55,000	<40,000
		C3	Pielou index of PC	>0.9	0.85–0.9	0.5–0.8	<0.5
		C4	Richness of PC	>8	6–8	5–6	<5
**B2**	**Soil Nutrients**	C5	SOM	>30g/kg	20-30g/kg	10-20g/kg	<6g/kg
		C6	TN	2g/kg	1.5g/kg	1g/kg	0.5g/kg
		C7	TP	2g/kg	1.5g/kg	1g/kg	0.5g/kg
		C8	TK	>20g/kg	10-20g/kg	5-10g/kg	<5g/kg
		C9	pH value	6–7	4.5–6; 7–8	3.5–4.5; 8–8.5	3–3.5; 8.5–9
**B3**	**Soil physical properties**	C10	Soil saturation water holding capacity	1–0.95%	0.95–0.9%	0.9–0.85%	0.85–0.8%
		C11	Soil moisture content	18.5% - 20%	15.5% - 18.5%	12%-15%	<8%
		C12	Soil bulk density	1–1.15g/cm^3^	1.15–1.35g/cm^3^	1.35–1.4g/cm^3^	1.4–1.7g/cm^3^
**B4**	**Ecological factors**	C13	PC Sustainability	8	6	4	2
		C14	Ecological protection function of PC	8	6	4	2

The evaluation model, evaluation index grading standards, literature review, two-by-two comparison of factors on the same level, and judgement matrix (constructed using a 1–9 scale method) were combined with the fuzzy comprehensive evaluation method. The index affiliation degree and fuzzy evaluation of the matrix evaluation weights for the weighted calculations resulted in four evaluation result grades (excellent, good, normal, and poor). Therefore, the evaluated grades of the highway slope reclamation effects were classified into four categories.

## Results

### Plant community characteristics

As demonstrated in Table 4, the plant community with the highest Shannon-Weiner index value was Guandi *A*. *fruticosa + Artemisia annua* (PC 2), which was mainly dominated by the shrub *A*. *fruticosa* and an annual herbaceous plant *A*. *annua*. *Setaria viridis*, *Digitaria sanguinalis* (L.) Scop, *Artemisia argyi H*. *Lév*. *& Vaniot*, and *Erigeron annuus* (L.) Pers, which included many herbaceous species in this plant community and 100% cover, also had a high SW index. The lowest SW index was observed for the Yuandi *Broussonetia papyrifera* community (PC 8), which included mainly trees and herbaceous cover below 50% for the whole community, and it also presented the lowest evenness index and species richness of the 10 communities. PC 8 showed poor overall recovery of the plant community and significantly lower community biomass relative to all the other communities. The community of Zhangdi *A*. *fruticosa + Periploca sepium* (PC 5) had the highest evenness index, low plant richness, few herbs, and only two herbaceous plant species (*A*. *argyi* and *Kali collinum* (Pall.) Akhani & Roalson), which presented a low cover level. The community with the highest productivity value was Zhangdi *Ulmus pumila + A*. *fruticosa* (PC 7), which was dominated by trees (*U*. *pumila*) and shrubs (*A*. *fruticosa*), while that with the lowest productivity was Yuandi *S*. *viridis + A*. *fruticosa* (PC 10), which was dominated by two herbaceous plants (*S*. *viridis* and *D*. *sanguinalis*) and included a small number of other species, mainly *E*. *annuus*, *K*. *collinum*, and *A*. *annua*. PC 10 presented a shrub layer with a low density of *A*. *fruticosa*, no trees, and low productivity.

**Table 4:** Plant community diversity indicators.

Number of PC	Shannon-Weiner index	Pielou index	Richness Index	PC productivity
**PC 1**	1.38±0.63	0.86±0.06	7.33±1.15	904219.56±282982.11
**PC 2**	1.85±0.11	0.85±0.04	9.00±1.00	259543.26±446274.13
**PC 3**	1.49±0.39	0.88±0.07	5.67±2.08	289987.52±475868.01
**PC 4**	1.27±0.16	0.87±0.03	4.33±0.58	1107.81±209.53
**PC 5**	0.93±0.44	0.94±0.05	3.0±1.73	4664.86±6128.70
**PC 6**	1.35±0.22	0.89±0.12	5.0±2.0	42028.94±47072.28
**PC 7**	1.24±0.12	0.71±0.03	5.67±0.58	977869.87±778144.52
**PC 8**	0.65±0.21	0.62±0.09	3.00±1.00	10150.35±4279.14
**PC 9**	1.55±0.02	0.86±0.02	6.0±0.00	6825.80±7989.73
**PC 10**	1.30±0.23	0.69±0.04	6.67±1.53	1085.34±1411.41

### Soil fertility

Among the soil chemical property indicators of the plant communities, the highest TN content was found in PC 5, and the lowest was in PC 8; the highest TP was in PC 3, and the lowest was in PC 8; the highest TK was in PC 6, and the lowest was in PC 10; and the highest SOM content was in PC 10, and the lowest was in PC 7. The pH of all 10 plant communities was alkaline and did not vary between communities (Table 5).

**Table 5:** Soil chemical property indicators.

Number of PC	TN (g/kg)	TP (g/kg)	TK (g/kg)	pH	SOM (g/kg)
**PC 1**	0.37±0.17	0.48±0.02	17.49±0.30	9.07±0.17	9.29±3.35
**PC 2**	0.35±0.05	0.48±0.02	17.38±0.04	8.99±0.11	9.41±1.58
**PC 3**	0.50±0.02	0.61±0.01	17.22±0.22	8.98±0.09	12.84±1.33
**PC 4**	0.41±0.07	0.53±0.05	17.88±0.29	8.92±0.05	8.81±1.43
**PC 5**	0.55±0.10	0.52±0.00	17.85±0.09	8.86±0.04	13.78±3.28
**PC 6**	0.35±0.18	0.48±0.05	18.37±0.41	9.00±0.14	8.01±3.99
**PC 7**	0.36±0.08	0.49±0.04	17.93±0.58	8.98±0.02	7.79±1.61
**PC 8**	0.33±0.03	0.47±0.03	17.70±0.28	8.94±0.03	8.61±1.53
**PC 9**	0.41±0.09	0.55±0.04	17.14±0.41	8.92±0.10	10.97±1.64
**PC 10**	0.52±0.12	0.54±0.01	17.12±0.47	8.97±0.02	14.16±4.60

### Soil physical properties

The mean values of the soil physical property indexes of the highway slope plant communities revealed that the highest soil saturation water holding capacity occurred in PC 9, and the lowest occurred in PC 7. The soil water content varied greatly among the 10 communities, with the highest in PC 10 and the lowest in PC 8. The highest soil capacity was in PC 7, and the lowest was in PC 3 (Table 6).

**Table 6:** Soil physical property indicators.

Number of PC	Soil saturation water holding capacity (%)	Soil moisture content (%)	Soil bulk density (g/cm^3^)
**PC 1**	1.09±0.03	6.94±0.72	1.31±0.03
**PC 2**	1.10±0.02	5.92±1.02	1.27±0.03
**PC 3**	1.26±0.15	9.60±1.04	1.17±0.14
**PC 4**	0.99±0.02	10.15±4.30	1.44±0.04
**PC 5**	0.97±0.01	8.78±0.71	1.45±0.01
**PC 6**	0.95±0.03	8.88±1.67	1.48±0.04
**PC 7**	0.90±0.03	4.82±0.23	1.54±0.04
**PC 8**	1.00±0.01	3.43±1.37	1.38±0.02
**PC 9**	4.29±5.62	10.92±1.42	1.33±0.06
**PC 10**	1.19±0.04	17.88±1.20	1.25±0.03

### AHP–fuzzy comprehensive evaluation of the slope reclamation effect

The field research results were combined with the AHP–fuzzy comprehensive evaluation method. The grade of the slope reclamation effect was obtained for all sampled plant community reclamation areas on the slopes of the Beijing-Hong Kong-Macao Expressway. The grades revealed that four PCs were classified as excellent while six sites were concentrated in other three grades (i.e., good, normal, and poor), thus indicating that further improvement is required. The reclamation effect of the three sample sites in the Guandi section was great, two PCs were excellent, one PC reclamation effect was good. Two PCs in the Zhangdi section were classified as excellent, and two PCs were classified as good and normal, resulting in a good overall reclamation effect. The reclamation effect of PCs in the Yuandi section were the worst, two of three PCs were evaluated as poor. According to the principle of maximum affiliation [36], the descending order of the slope reclamation effect evaluation was as follows: PC 7 > PC 2 > PC 1 > PC 6 > PC 3 > PC 9 > PC 5 > PC4 > PC 8 > PC 10 (Table 7).

**Table 7:** AHP–fuzzy comprehensive evaluation.

Number of PC	Place name	Excellent	Good	Normal	Poor	Evaluation results
**PC 1**	Guandi	0.34	0.26	0.12	0.28	Excellent
**PC 2**	Guandi	0.42	0.35	0.07	0.14	Excellent
**PC 3**	Guandi	0.28	0.41	0.09	0.22	Good
**PC 4**	Zhangdi	0.34	0.17	0.98	0.40	Normal
**PC 5**	Zhangdi	0.30	0.33	0.16	0.21	Good
**PC 6**	Zhangdi	0.33	0.24	0.17	0.27	Excellent
**PC 7**	Zhangdi	0.53	0.10	0.21	0.15	Excellent
**PC 8**	Yuandi	0.25	0.12	0.14	0.5	Poor
**PC 9**	Yuandi	0.25	0.36	0.16	0.21	Good
**PC 10**	Yuandi	0.08	0.20	0.17	0.33	Poor

## Discussion

Artificially restored plant can provide the groundwork for the natural recovery of slope plant, shorten the early plant community succession duration, and effectively improve slope soil [37], thus creating the conditions required for restoring the original ecological environment. The slopes of the Beijing-Hong Kong-Macao Expressway were restored over 20 years ago. The early reclamation only included *A*. *fruticosa*, which has become established and remains the dominant species. However, the community composition of the restored ecosystems differed from that of the target plant community, thus revealing that selecting early plant species is particularly important.

### Evaluation of plant community reclamation effect

Based on the result of this research, most of the 10 PCs which have been growing for more than 20 years have good evaluation of reclamation. Another research of us showed that most of the PCs that have been growing for 9 years were normal, some of which were poor [38]. This result suggests that time is important for ecological restoration. With the increase of time, both species diversity and composition have improved [39]. In order to better understand the characteristics of slope PCs, we analyzed the four excellent PCs. The mulberry family (mainly *B*. *papyrifera*) has the most plants, followed by Leguminosae, Asteraceae, and Gramineae. The main plant species were *Cynodon dactylon (L*.*) Pers*, *D*. *sanguinalis*, *A*. *annua*, *A*. *fruticosa*, and *Lespedeza bicolor* Turcz. All of these excellent PCs had a tree–shrub–grass community structure, thus demonstrating that richly layered communities are more effective in water and soil conservation [40]. PC6 had the highest family richness.which included 8 families and 9 genera, and it was dominated by *B*. *papyrifera* and the shrub *A*. *fruticosa*. *Broussonetia papyrifera* has a well-developed and deep root system and thus can effectively absorb soil nutrients, and it is also fast-growing and highly adaptable. The shrub *A*. *fruticosa* is a shade-tolerant and saline-tolerant perennial that can rapidly achieve community reclamation. A common feature of the excellent PCs was the abundance of leguminous plants, which can effectively improve soil fertility [41]. All the PCs with a relatively high species diversity index contained *A*. *annua*. Although *A*. *annua* is an annual herb, it has various advantages when used in different community configurations. In addition, the plant in these communities included vigorous species belonging to the family Asteraceae that are capable of rapidly covering a given area and lack strict requirements for soil texture. Thus, these species are used to restore steep and high roadbed slopes. When restoring steep and high roadside slopes with intense sunshine or poor soil quality, *A*. *annua* is often recommended. Therefore, when restoring slopes, the proportion of legumes, Asteraceae, and Gramineae among the sprayed grass seeds should be high to accelerate community reclamation. These findings are consistent with previous research [42–44], which suggested that the Plant community composition and structure of roadbed slopes should include a combination of trees, shrubs, and grasses and a high proportion of legumes and daisies planted in combination with native species to ensure that the slope is restored quickly. There are less Asteraceae, Leguminosae, and Gramineae in the remaining six PCs. These plant community combinations were too homogeneous and included tree-grass structures or shrub–grass structures, which are not conducive to effective side slope plant community reclamation [45]. This was especially evident in PC 10, which was dominated by herbs and had the worst reclamation effect. This finding is consistent with the evaluation results. We also noticed that even with the same early plants, the slope has developed differernt plant community over time. Plant—soil interactions play an important role in causing changes in species compositon, this point was consistent with Mosanghini D et al’s research [46].

### Evaluation of soil physicochemical properties

Although the relationship between soil fertility and community reclamation has been previously studied, varying results were obtained, with most studies suggesting that the SOM and TN contents of soil play an important positive role in the reclamation of slope communities. The highest contents of TN and SOM were found in PC 3, PC 5, and PC 10. A total of 99% of the TN content was from SOM [47, 48], indicating that SOM and TN are the main factors impacting the slope reclamation effect. Although the SOM and TN contents of PC 3, PC 5, and PC 10 were the highest among the 10 PCs, the values were still low based on soil standards. According to the 2nd Chinese soil nutrient grading standard, the TN content of these research areas was at level 5, and SOM content was at level 4, which are considered deficient. The soil nutrients in the remaining communities correspond to grades of normal and low grades, and the result shows that the overall soil nutrient level along these research slopes was low. Soil bulk density directly affects the soil and is an important index of soil physical properties. As an important indicator of soil physical properties, soil bulk density directly affects the ratio of water to air. If the soil bulk density is too high, then soil permeability will be poor, and plant root growth will be affected. If the soil bulk density is too low, then the soil texture will be loose, and the uptake and decomposition of plant organic matter will be affected [49–51]. In PC 4, PC 5, PC 6, and PC 7, the soil bulk density values were all above 1.4 g /cm^3^, and the soil moisture contents were low at below 10%. This finding may be related to the main plants found in all four PCs, which are trees and shrubs; the 0–20-cm soil layer is compact because trees and shrubs have highly developed root systems with higher root mass in deeper soil layers [52]. Soil quality negatively correlates with recovery time, with a longer recovery duration corresponding to a lower soil quality. Because of the costs associated with maintenance, slope soil improvements are usually only performed at the initial stage of artificial slope construction and include the spraying of mixed fertilizers over the slopes to improve the soil. Over time, improvements in soil quality are thus totally dependent on the plant community; therefore, early plant species selection is particularly important.

### Problems and recovery options

#### Species selection

At the early stage of artificial planting, plant species should be selected based on the conditions of the various sites. Many scholars recommend the use of native species because they can adapt to new environments. However, highway construction destroys the original habitat; thus, previously dominant pioneer species may not easily adapt to the new site conditions, which will prolong the natural succession duration and affect the natural slope reclamation process [53]. Research has indicated that planting various combinations of native and exotic species represents an efficient method of ecologically restoring slopes because such communities may be capable of rapidly colonizing and covering the site in the early reclamation stages, which would effectively ensure community stability [54]. Our research reveals the best plant community composition and structure for highway slopes is a combination of trees, shrubs, and herbs, we recommend *B*. *papyrifera* / *U*. *pumila* + *A*. *fruticosa* / *L*. *bicolor* + perennial herbs (e.g., *C*. *dactylon* / *Oxalis corniculata* L / *Equisetum ramosissimum* Desf / *Medicago sativa* L.). Boscutti F. et al’s research also indicated that using perennial herbs and trees can promote succession [55]. Trees such as *B*. *papyrifera* and *U*. *pumila*, which have developed root systems, fast growth rates, and strong adaptability, are excellent fast-growing trees for soil fixation and preservation and drought resistance. However, the highway maintenance department often avoids planting trees because they may cause lodging, scrape vehicles, and introduce other safety risks. Considering that tree root systems have a good anchoring effect and thus can improve slope stability, suggested planting trees at the lower slope [56, 57]. We suggest that trees should be planted at lower fill slopes. Shrubs such as *A*. *fruticosa* and *L*. *bicolor* present dense foliage and broad cover, and they improve soil and can adapt to soil conditions in habitats with varying climatic conditions. Pioneer herbaceous vegetation, such as *C*. *dactylon* and *O*. *corniculate*, can quickly cover slopes in the early stages of slope reclamation and are capable of achieving 60% cover in a short time (Wang et al., 2021). In the early stage of slope reclamation, selecting the identified plant species for mixed seeding may more effectively prevent the invasion of strangler plants (such as *Humulus scandens*) and assist the recovery of the slope plant community to enable natural succession in future stages.

#### Soil improvement

The results revealed that even in the excellent PCs, the pH value of the soil was weakly alkaline, which may limit plant growth. Engineering methods are available to improve the initial soil quality, such as using mulch to add organic nutrients, which would need to be adjusted to the appropriate level for each specific site [58]. This research revealed that for highway slopes with long recovery times, species diversity should be enhanced because such diversity is the key to improving soil [41]. Therefore, selecting native plants, such as legumes, Graminaceae, and Chrysanthemums (such as *A*. *fruticosa*, *L*. *bicolor*, *D*. *sanguinalis*, and *C*. *dactylon*), represents a good choice for improving slope soil [9].

## Conclusion

We analyzed the species diversity and soil physicochemical indexes of 10 representative PCs on the slopes of the Zheng-Xin section of the Beijing-Hong Kong-Macao Expressway after 23 years of reclamation. The data were combined with the results of an ecological protection function questionnaire, and the AHP–fuzzy comprehensive evaluation method was used to perform a reclamation evaluation. The result showed that the reclamation evaluation of research road was good, but a few PCs still need to be improved. Based on the evaluation, plant community composition and structure that is mainly composed of trees (*B*. *papyrifera* or *U*. *pumila*), shrubs (with *A*. *fruticosa* as the main dominant species), and herbs (such as *A*. *annua* and *C*. *dactylon* as the main herbs) would be good choice for highway slopes. This research could provide method of ecological restoration for highway slope.

## Supporting information

S1 Data (XLSX)
